# Effect of the Movement Control Order on the Incidence of Complicated Appendicitis During the COVID-19 Pandemic: A Cross-Sectional Study

**DOI:** 10.21315/mjms2021.28.5.13

**Published:** 2021-10-26

**Authors:** Hamzah Sukiman, Abdul Malek Mohamad, Muhammad Firdaus Nasution Raduan, Mohd Nur Afiq Mohd Yasim, Muhammad Ikhwan Mat Lazim

**Affiliations:** 1 Department of Surgery, Hospital Tengku Ampuan Afzan, Kuantan, Pahang, Malaysia; 2 Department of Surgery, International Islamic University Malaysia Medical Centre, Kuantan, Pahang, Malaysia; 3 Department of Surgery, Hospital Sultan Haji Ahmad Shah, Temerloh, Pahang, Malaysia; 4 Hospital Pekan, Pekan, Pahang, Malaysia

**Keywords:** Movement Control Order, COVID-19, appendicitis

## Abstract

**Background:**

Appendicitis is common and delayed presentation results in complicated appendicitis with increased morbidity. This study investigates the effect of the Movement Control Order (MCO) during the COVID-19 pandemic on the presentation and severity of appendicitis.

**Methods:**

A cross-sectional study including 193 patients diagnosed with appendicitis was conducted at four hospitals in Pahang, Malaysia. Those who presented between 1 February 2020 and 17 March 2020 were included in the pre-MCO group and those between 18 March 2020 and 30 April 2020 in the MCO group. The definitions of simple and complicated appendicitis were based on the Sunshine Appendicitis Grading Score. The primary outcome was the incidence of complicated appendicitis, and the secondary outcomes were length of stay, a composite of surgical morbidities and a composite of organ failure.

**Results:**

A total of 105 patients in the pre-MCO group and 88 in the MCO group were analysed. The incidence of complicated appendicitis was 33% and it was higher in the MCO than in the pre-MCO group (44% versus 23%, *P* = 0.002). The MCO period was independently associated with complicated appendicitis in the logistic regression (*P* = 0.001). It was also associated with prolonged length of stay (3.5 days versus 2.4 days, *P* < 0.001) and higher overall surgical morbidity (19% versus 5%, *P* = 0.002).

**Conclusion:**

The MCO imposed during the COVID-19 pandemic was associated with a higher incidence of complicated appendicitis and surgical morbidity.

## Introduction

Acute appendicitis is the most common surgical emergency encountered in general surgical practice (1). The disease may present as a simple inflammation of the appendix, or it may be complicated by perforation with resultant purulent or feculent contamination, which is associated with higher morbidity. Appendicectomy is the treatment of choice, with most patients recovering without complications, although a non-operative approach is a viable choice for a certain subset of the patient population. Early presentation, diagnosis and treatment are crucial for reducing surgical morbidities related to complicated appendicitis.

The world was shocked by a new highly contagious respiratory disease resulting from a novel coronavirus, which was initially reported in Wuhan, China in late 2019 and has since been named COVID-19 (2). The disease spread globally, and in March 2020 the World Health Organization (WHO) declared a pandemic. This led to a chain of events that finally resulted in the enforcement of a nationwide partial lockdown in Malaysia, termed the Movement Control Order (MCO), starting on 18 March 2020 and continuing well into May 2020 in its fifth iteration.

Although movement with the intent to seek treatment is exempted from the MCO, such measures may discourage the public from seeking treatment at the early stage of the disease. It is possible that patients only sought treatment once the symptoms became unbearable, causing delays in diagnosis and treatment. This study aimed to verify whether patients presented with appendicitis in the complicated stage during the MCO and to determine the outcome associated with such a presentation.

While the diagnosis of appendicitis is clinical, it can be aided by diagnostic scoring systems such as the Alvarado score (3), which has been validated and found to be reliable for the diagnosis of appendicitis. However, its use in predicting the severity of appendicitis remains controversial. A study on a large Polish cohort showed that a higher Alvarado score is associated with a risk of developing complicated appendicitis (4). This finding was corroborated by another study by Imaoka et al., which incorporated the use of a temperature > 37.4 °C, C-reactive protein (CRP) > 4 and the presence of periappendiceal fluid as important indicators for predicting complicated appendicitis (5).

Appendicitis can be classified based on its severity as either simple or complicated. Multiple grading systems utilising intraoperative findings are used, such as the Sunshine appendicitis grading system score (SAGS) (6) and the scoring system proposed by the World Society of Emergency Surgery (WSES) (1). The SAGS score is validated for the intraoperative diagnosis of simple and complicated appendicitis, with good inter-rater agreement (6).

A review of more than 900 appendectomies in a Malaysian general hospital revealed that the incidence of complicated appendicitis is 15% (7). Research on the risk factors for developing complicated appendicitis is still lacking, although age, female gender, obesity, diabetes mellitus, immunocompromised states, duration of symptoms, a higher Alvarado score and high CRP have been shown to be associated with complicated appendicitis (8). It is reasonable to postulate that presenting late to the hospital will lead to complicated appendicitis, which poses a higher mortality risk. Furthermore, outcomes such as perioperative morbidity, required re-interventions and length of stay are worse in complicated appendicitis (5).

The primary objective of this study was to determine whether there was a higher incidence of complicated appendicitis in the MCO period during the COVID-19 pandemic. Secondary objectives included determining other risk factors associated with complicated appendicitis and measuring the adverse outcomes associated with complicated appendicitis (increased length of stay, surgical morbidity and organ failure).

## Methods

A cross-sectional study including 193 patients was conducted at four hospitals providing general surgical services in Pahang, Malaysia between February and April 2020. We included all patients admitted with a clinical diagnosis of appendicitis, made either by (i) suggestive history and physical examination by a specialist in general or paediatric surgery; (ii) imaging findings (ultrasound or CT scan) suggestive of appendicitis; or (iii) intraoperative findings of appendicitis. Patients for whom an initial diagnosis of appendicitis was made but were subsequently treated for other causes of abdominal pain were excluded.

Universal sampling was employed, with patients presenting between 1 February and 17 March 2020, before the MCO was implemented, grouped into the ‘pre-MCO’ group. Those presenting between 18 March and 30 April 2020 were grouped into the ‘MCO’ group. Presentation beyond 48 h after the onset of symptoms was considered as delayed presentation.

Patients were followed up from admission up to the point of discharge. Demographic and clinical data were obtained from patients’ records, and a comparison was made between the pre-MCO and MCO groups. The primary outcome of interest was the incidence of complicated appendicitis, and secondary outcomes were length of hospital stay, a composite of surgical morbidities and a composite of organ failure requiring support.

Simple appendicitis is defined as appendicitis that resolves with non-operative management or cases graded intraoperatively as grades 0–1 based on the SAGS score. Complicated appendicitis is defined as appendicitis graded intraoperatively as grades 2–4 based on the SAGS score and those who failed non-operative management and subsequently graded 2–4 during surgery.

The sample size was calculated as follows:


$$n=\frac{{\left({\text{z}}_{1-\alpha /2}\right)}^{2}\;(P)\;(q)}{{\left(d\right)}^{2}}=195$$

where Z_1−α/2_ value is taken at 1.96 at a 95% CI and a 5% level of significance

*P* = 15% incidence from previous study
*q* = 1–*P*
*d* = Margin of error or precision taken at 0.05

The collected data were analysed using IBM SPSS Statistics version 23. Categorical variables were expressed as frequencies and percentages. Numerical data were considered normally distributed if the mean was equal to the median and they had skewness and kurtosis values between +2 and −2. The data were presented as means and standard deviations if they were normally distributed and as medians and interquartile ranges (IQR) if not normally distributed.

Univariate analyses of the associations between categorical variables (gender, immunocompromised states, pre-operative organ failure, surgical access, antibiotics and surgical morbidity) were performed using the Fisher’s exact and chi-squared tests. The Mann–Whitney U (for non-normally distributed data) and independent sample T (for normally distributed data) tests were used for the numerical variables (age, BMI, Alvarado score, duration of surgery and length of stay). A *P*-value of 0.05 was considered the threshold for statistical significance. Analysis using binary logistic regression was also performed to determine whether the MCO period was independently associated with an increased incidence of complicated appendicitis.

Application for ethical approval was submitted to the Malaysian Research Ethics Committee (MREC).

## Results

A total of 229 patients were screened for eligibility, and after excluding 36 patients, 193 were included in the final analysis. Among the 193 patients included in the final analysis, a total of 105 were included in the pre- MCO group and 88 in the MCO group. The demographics between the two groups were relatively homogenous. There was a slight female predominance (51%), and an overwhelming majority of patients (92%) did not have preexisting immunosuppressive states. Patients’ age ranged between 5 years old and 75 years old, with a mean of 29 years old. Paediatric patients constituted a minority of our study population (*n* = 14, 7.3%). Most patients (67%) lived within the same district as the hospital they presented to (Table 1).

In total, 63 (33%) patients had complicated appendicitis, defined as a SAGS score of 2–4. Thirty-one patients (16%) were treated successfully with non-operative management and were considered to have simple appendicitis (Table 2).

Analysis using a chi-squared test showed that the MCO period was associated with a significantly higher incidence of complicated appendicitis (*P* = 0.002). Patients presenting during the MCO period had delayed presentation, with a mean duration of symptoms of 2.6 days versus 2.0 days in the pre-MCO group (*P* = 0.022). They also had clinical features correlating with higher Alvarado scores, with a median score of 7 versus a score of 6 in the pre-MCO group (*P* = 0.012). The incidence of preoperative organ failure was also higher in the MCO group, although it did not reach a level of statistical significance (Table 2).

A total of 7% of the patients with appendicitis who presented during the MCO period required laparotomy, compared to 4% in the pre-MCO group (*P* = 0.019). A total of 31 patients were successfully treated with antibiotics, without needing surgery. There was also a higher proportion of patients receiving antibiotics for an extended duration in the MCO group (16% versus 4%, *P* < 0.001). The incidence of a composite of surgical morbidities was also higher in the MCO group (*P* = 0.002) and these patients had a longer duration of hospitalisation (*P* < 0.001). All patients who developed organ failure requiring support were in the MCO group, although it was a small number that did not reach the threshold for statistical significance (*P* = 0.087) (Table 3).

In the simple logistic regression, duration of symptoms (odds ratio [OR] 1.204; 95% CI 1.031–1.407; *P* = 0.019) and the MCO period (OR 3.573; 95% CI 1.659–7.694; *P* = 0.001) were found to be independently associated with complicated appendicitis (Table 4).

## Discussion

There have been many challenges in managing surgical emergencies during the COVID-19 pandemic, the foremost of which is ensuring a timely diagnosis and intervention. Appendicitis remains the most common surgical emergency in surgical practices worldwide (7). Patients with simple appendicitis can typically be managed with a single dose of antibiotics and appendicectomy, rarely requiring admissions of more than 48 h. In contrast, a perforated appendix can be associated with a myriad of complications. This results in prolonged hospital stay and increased morbidity and imposes a further burden on the healthcare system.

Our study demonstrated an independent association between the MCO period and incidence of complicated appendicitis. The MCO imposed a significant limitation on human movement hitherto unseen in this century. A possible explanation for the increased incidence of complicated appendicitis is reluctance on the part of patients to seek early medical attention upon developing symptoms. This may be due to the general difficulty of moving around during the MCO period or the mistaken perception that specialist hospitals currently deal only with COVID-19 cases. The hypothesis of patients presenting in later stages during the MCO period is supported by the significant difference observed in the duration of symptoms between the pre-MCO and MCO groups.

Neither age, BMI, distance to hospital, nor pre-existing immunocompromised states were associated with the development of complicated appendicitis. Complicated appendicitis was more prevalent among male patients in our study population. Augustin et al. (9) observed that the duration to perforation is significantly shorter in males, resulting in complicated appendicitis at presentation.

There was a higher chance that patients with appendicitis presenting during the MCO period would have a higher Alvarado score and organ failure, which we took as proxy markers of disease severity at presentation and were correlated with the histologic findings. This was consistent with previous works published by other authors (10). Organ failure also complicates subsequent patient recovery during the post-operative period.

Patients presenting during the MCO period were more likely to require a laparotomy, as opposed to the much smaller surgical access required for the Lanz incision. This was likely due to the clinical finding of generalised peritonitis during the pre-operative assessment, denoting a perforated appendix. They were also more likely to require prolonged antibiotic treatment, which may indicate persistent intra-abdominal sepsis or a recovery complicated by superimposed nosocomial infection.

The overall complication rate was 11%, in line with rates of 3% to 29% reported in literature (1). The MCO group was more likely to develop various post-operative complications based on our analysis of a composite of surgical morbidities, with the most common being post-operative ileus and surgical site infection.

The COVID-19 pandemic has placed a strain on health care resources and exposed health care workers to occupational hazards. In light of this, the College of Surgeons, Academy of Medicine of Malaysia has published an advisory which advocates the use of a non-operative approach for conditions such as appendicectomy. However, for such approach to be possible, it is imperative that patients present at an early stage, without appendiceal perforation and generalised peritonitis, which require operative intervention.

### Limitations and Recommendations

COVID-19 is a new disease and its full impact on people’s social norms and health-seeking behaviour has not yet been fully elucidated. This study is limited to describing the association between the MCO and the presentation of complicated appendicitis, which may or may not imply causation. A larger, population-based study comparing two or more localities with differing degrees of movement restriction would better quantify the strength of this association.

## Conclusion

The MCO period was associated with an increased incidence of complicated appendicitis, resulting in higher rates of surgical morbidities and prolonged length of hospital stay. During the COVID-19 pandemic, common conditions like appendicitis must be managed in an expeditious and efficient manner, both to ensure minimal patient morbidity and to optimise the use of resources. However, the MCO appears to have had an inadvertent effect of causing patients to present with complicated appendicitis.

## Figures and Tables

**Table 1:** Demographic characteristics of patients with appendicitis (*n* = 193)

Variables	All patients (*n* = 193)	Pre-MCO (*n* = 105)	MCO (*n* = 88)	*P*-value
Age (years old)	29 (15)	27 (14)	31 (15)	0.307
Gender				
Male	94 (49)	46 (44)	48 (55)	0.137
Female	99 (51)	59 (56)	40 (45)	
Immunocompromised state	15 (7.8)	9 (8.6)	6 (6.8)	0.650
Diabetes mellitus	13 (6.7)	8 (7.6)	5 (5.7)	0.593
Steroid therapy	5 (2.6)	3 (2.9)	2 (2.3)	0.583
No immunocompromised state	178 (92)	96 (91)	82 (93)	
Place of residence				
Within district	129 (67)	69 (66)	60 (68)	
Out of district	64 (33)	36 (34)	28 (32)	0.717

Notes: Numerical data are expressed as means (SD). Categorical data are expressed as frequencies (percentages)

**Table 2:** Clinical presentation and diagnosis

Variables	All patients (*n* = 193)	Pre-MCO (*n* = 105)	MCO (*n* = 88)	*P*-value
Simple appendicitis	130 (67)	81 (77)	49 (56)	
Complicated appendicitis	63 (33)	24 (23)	39 (44)	0.002
Duration of symptoms (day)	2.3 (2.1)	2.0 (2.1)	2.6 (2.1)	0.022
Alvarado score	6 (2)	6 (2)	7 (2)	0.012
Pre-operative organ failure (comorbidities)	16 (9)	5 (5)	11 (13)	0.052
Shock	6 (3)	1 (1)	5 (5.7)	0.070
Acute kidney injury	10 (5.2)	4 (3.8)	6 (6.8)	0.269
Respiratory failure	1 (0.5)	0 (0)	1 (1)	0.456
No organ failure	177 (91)	100 (95)	77 (87)	

Notes: Duration of symptoms is expressed as means (SD) and Alvarado scores are expressed as medians (IQR). Categorical data are expressed as frequencies (percentages)

**Table 3:** Management and outcome

Variables	All patients (*n* = 193)	Pre-MCO (*n* = 105)	MCO (*n* = 88)	*P*-value
Surgical access				0.019
Laparoscopic	17 (9)	15 (14)	2 (2)	
Lanz incision	135 (70)	76 (72)	59 (67)	
Laparotomy	10 (5)	4 (4)	6 (7)	
Non-operative	31 (16)	10 (10)	21 (24)	
Antibiotics				
Prophylactic	83 (43)	62 (58)	21 (24)	< 0.001
Treatment (≤ 7 days)	92 (48)	39 (37)	53 (60)	
Extended (> 7 days)	18 (9)	4 (4)	14 (16)	
Duration of surgery (min)	59 (40)	53 (28)	61 (35)	0.067
Length of stay (day)	2.9 (3.4)	2.4 (2)	3.5 (4.2)	< 0.001
Surgical morbidity	22 (11)	5 (5)	17 (19)	0.002
SSI/wound dehiscence	7 (4)	1 (1)	6 (7)	
Intraabdominal collection	1 (0.5)	0	1 (1)	
Re-operation	1 (0.5)	0	1 (1)	
Post-operative ileus	12 (6)	2 (2)	10 (11)	
Intestinal obstruction/perforation	1 (0.5)	0	1 (1)	
Cardiac complications	2 (1)	2 (2)	0	
Respiratory complications	5 (3)	1 (1)	4 (5)	
Organ failure requiring support	3 (1.7)	0	3 (3.8)	0.087
Mechanical ventilation	3 (1.6)	0	3 (3.4)	
ICU admission	3 (1.6)	0	3 (3.4)	
Inotropic support	2 (1)	0	2 (2.3)	

Notes: Duration of surgery is expressed as median (IQR) and length of stay as mean (SD). Categorical data are expressed as frequencies (percentages)

**Table 4:** Simple logistic regression analysis of risk factors for complicated appendicitis

Parameters	Wald’s χ^2^	*P*	Crude OR	OR estimates (95% CI limits)	

				Lower	Upper
Age	0.206	0.650	1.006	0.980	1.033
Gender (male)	9.606	0.002	0.301	0.141	0.643
Immunocompromised state	0.145	0.703	1.319	0.317	5.481
Duration of symptoms	5.471	0.019	1.204	1.031	1.407
Living within district	0.594	0.441	0.722	0.315	1.654
MCO period	10.589	0.001	3.573	1.659	7.694
